# Factors influencing chopstick use and an objective identification of traditional holding techniques in children

**DOI:** 10.1371/journal.pone.0314113

**Published:** 2025-01-09

**Authors:** Yuki Choji, Nanami Hirokawa, Chie Morimoto, Norihito China, Akio Nakai, Kazunori Miyata

**Affiliations:** 1 Occupational Therapy Course, Department of Rehabilitation, Faculty of Allied Health Science, Niigata University of Rehabilitation, Murakami, Japan; 2 Japan Advanced Institute of Science and Technology, Nomi, Japan; 3 Research Institute for Education & Graduate School of Clinical Education, Mukogawa Women’s University, Nishinomiya, Japan; Tsinghua University, CHINA

## Abstract

The acquisition of chopstick skills is considered essential for child development and etiquette in many Asian cultures. However, a decline in chopstick education has been observed in Japan, and the underlying causes of this phenomenon remain elusive. This study aims to investigate children’s chopstick skills and develop an objective method to evaluate them using a hand posture estimation model. In this study. A questionnaire survey was conducted among 165 first-grade elementary school students (aged 6–7) and their parents to investigate factors influencing chopstick proficiency. To complement this, video analyses were performed using a hand posture estimation model to assess the accuracy of chopstick grip classification. The findings showed that children’s chopstick-holding styles could be classified into four categories: four-finger prehension (64 participants), three-finger prehension (49 participants), palm prehension (20 participants), and others (32 participants). Despite the fact that over 80% of parents reported teaching their children how to use chopsticks, a mere 9.7% of children exhibited correct chopstick-holding technique. Interestingly, factors such as intergenerational cohabitation with grandparents and child’s age significantly influenced chopstick proficiency. These results indicate that a gap exists in the intergenerational transmission of chopstick skills, with parents potentially lacking sufficient knowledge to teach their children. The hand posture estimation model had a high accuracy rate of 85%, precision of 83%, and recall of 88% to identify whether children use chopsticks traditionally. While chopstick education is predominantly conducted within Japanese households, the increasing prevalence of nuclear families and dual-income households suggests a decline in intergenerational transmission of chopstick education. To address this issue, it is imperative to develop web applications that can integrate chopstick education into school curricula and promote chopstick skills among students.

## Introduction

Using chopsticks correctly is traditionally considered a developmental milestone for children in cultures where chopsticks are commonly used. Acquiring chopstick skills in early childhood activates the brain [1–3], enhances fine motor skills [4], and promotes good table manners. Moreover, studies have shown that chopstick use can reduce portion sizes, lower post-meal blood sugar levels, and help prevent obesity [5–7]. Furthermore, beyond developmental benefits, the popularity of Asian cuisine, known for its health benefits, has led to the widespread adoption of chopsticks in 33% of the world’s population [8]. Thus, chopsticks, alongside forks and spoons, have become indispensable tools in global dining practices.

With over 20 variations of chopstick grips [9], it is ideal for children to develop a grip that suits them. However, previous studies have primarily focused on comparing two types of chopstick-holding styles: the traditional pincers-pinching style and the scissors-pinching style, where the chopsticks are crossed between the finger Ⅰ and Ⅱ [10–12]. While the scissors-pinching style has been reported to provide a stronger grip, it has also been shown to be less precise compared to the traditional style. Although further research is needed to investigate the performance and precision of other holding styles, the traditional style is currently most recommended in terms of both operability and etiquette [12–14]. To hold chopsticks traditionally, begin by placing the lower chopstick, also known as the “fixed” chopstick, between fingers I and II, with the first knuckle of the finger Ⅳ supporting it. Afterward, place the upper chopstick, also known as the “movable” chopstick, alongside finger III and hold it between the tips of fingers I and II. Finally, use the “movable” chopstick to grasp food, while the “fixed” chopstick provides stability [10]. It is generally reported that children start using chopsticks around the age of 3 and become proficient by the age of 6 or 7 [15]. However, in Japan, the number of children who can traditionally hold chopsticks has been declining annually since around 1978 [16]. A 2010 survey by the Japan Cabinet Office [13] revealed that only 54.2% of adults (ages 18–70 years) can traditionally hold chopsticks. One reason for this decrease is that the influx of diverse foreign cuisines into Japan has influenced Japanese food culture and the use of traditional Japanese eating utensils, leading to a decrease in the frequency of chopstick use and a potential increase in the number of children with poor dexterity [4]. Additionally, a survey conducted by the Ministry of Agriculture, Forestry and Fisheries revealed that while 88.9% of respondents considered it is important for children to learn how to use chopsticks from their parents or communities, 52.7% of those in the 20–39 age group reported not having been taught by their parents or communities [17]. This suggests that the rise of nuclear families and dual-income households in Japan may have contributed to a decline in parental involvement in chopstick education. Although the exact causes of this decline are not fully understood, it is clear that factors beyond a child’s neural control and motor skills may significantly impact their ability to acquire chopstick skills.

It is essential to provide proper evaluation for children to master traditional chopstick usage. Traditional methods for assessing chopstick skills in children include measuring the time it takes to move simulated food, the force used to pinch food, and the electromyogram of the dominant hand while using chopsticks [10, 12, 18, 19]. In recent years, methods for evaluating chopstick manipulation in adults have been introduced that utilize OpenPose to estimate hand skeletal information or AR technology to provide feedback to users [20, 21]. However, while it is crucial for parents and children to learn and assess chopstick usage together, these methods often require a laboratory setting, which can be stressful for children and make it difficult to assess their skills in a real-world context. Hence, most evaluations of children’s chopstick skills are conducted through surveys targeting parents or children. According to Choji et al.’s review [14], while questionnaires are a convenient method for assessment, they have been reported to yield discrepancies between children’s and parents’ responses due to subjective reporting and may not accurately assess children’s chopstick skills. Therefore, objective evaluation of children’s chopstick handling is inadequate.

Considering the above, this study has a two-fold purpose. First, it aims to investigate the current state of children’s chopstick handling skills and explore the factors influencing the acquisition of traditional chopstick-holding techniques. Second, to propose an objective method for evaluating traditional chopstick-holding skills in children without the need for a conventional experimental setting, we introduce a hand posture estimation model that can be applied in a clinical setting.

## Methods

### Participants

A total of 165 first-grade students (75 boys, 90 girls; 114 aged 6, 51 aged 7) from 12 schools in Murakami City participated in this study. Participants were recruited through Murakami City Hall. Seven participants were excluded due to absence. The participant recruitment period was from May 12, 2023, to November 30, 2023. Handedness was assessed using the modified Edinburgh Handedness Inventory [22], revealing 138 right-handed, 12 left-handed, and 15 undetermined participants. The average length of the dominant hand was 133.50 ± 9.50 mm. Experimental procedures were explained in an age-appropriate manner, and written informed consent was obtained from both children and their parents. This study was approved by the Ethics Committee of Niigata Rehabilitation University (No. 235) and followed the Declaration of Helsinki.

### Materials and setup

**Fig 1:**
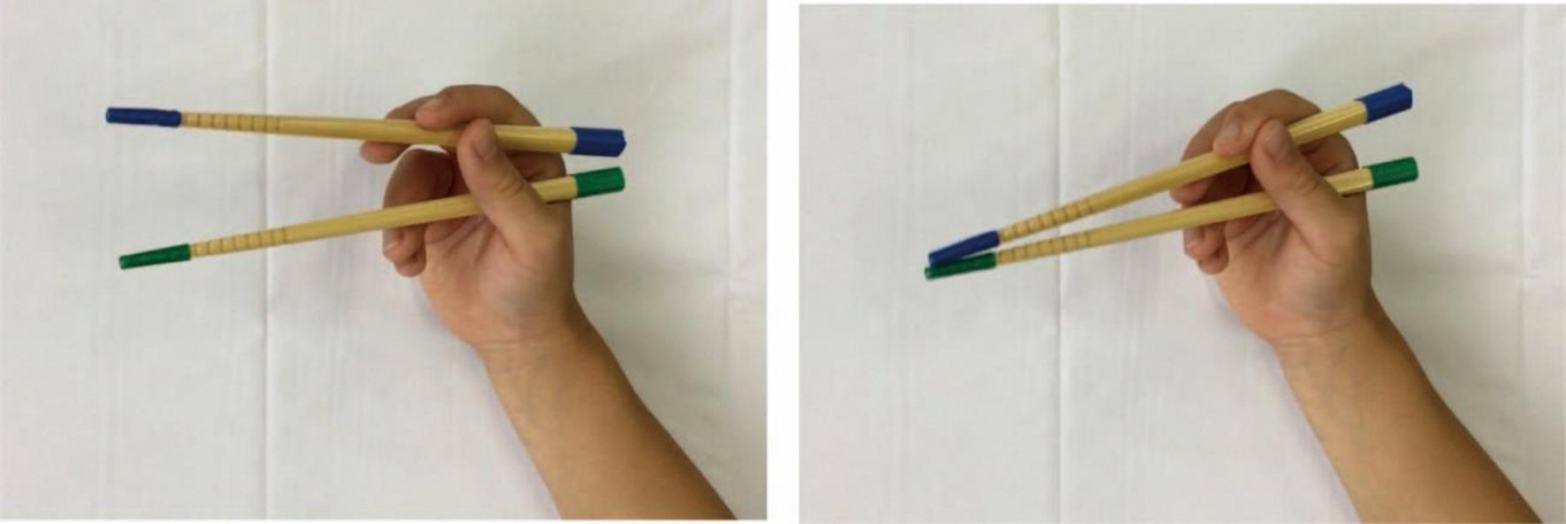
Chopstick opening and closing. The figure shows the traditional chopstick grip. Both ends of the upper and lower chopsticks are wrapped in blue and green tapes, respectively.

This study recorded the chopstick usage patterns of children using an iPad mini 4 (Apple Inc.). The device’s 7.9-inch Retina display, A8 chip, and 8MP rear camera were used to capture high-quality video footage. The video shooting occurred in the general classrooms of each elementary school, where tables and chairs that matched the body types of the children were provided. The blue and green tape was wrapped around the ends of the bamboo chopsticks to help identify and detect children’s hands and chopsticks against the background (Anzen Hashi, KYOEI Co., Hiroshima), and a white cloth was placed on the table (Fig 1).

**Fig 2:**
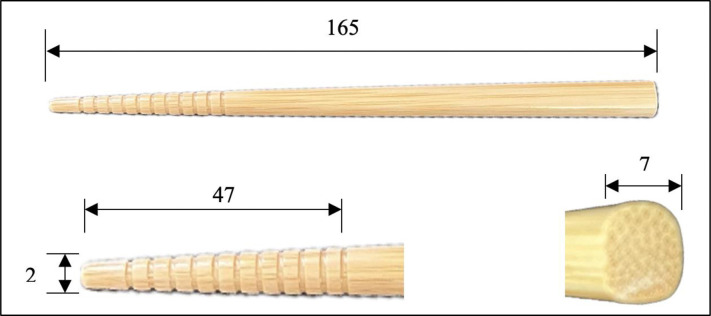
Traditional way to hold chopsticks. The length is in millimeters (mm).

The chopsticks described have a square handle with a diameter of 7 mm, a tip diameter of 2 mm, a weight of 8 g, and a length of 165 mm. Additionally, the tip has a groove (Fig 2). Traditional reports from China recommended that the most suitable chopsticks have a round handle with a diameter of 6 mm, a tip diameter of 4 mm, and a length of 18 cm [10, 18, 23]. In contrast, Japanese chopsticks vary from Chinese chopsticks because of differences in dietary culture. Most chopsticks sold in the Japanese market are square-handled, with a diameter of 6–7 mm at the handle, 2–3 mm at the tip, and a weight of >8 g. The ideal length of chopsticks is considered 1.2 times the length of the hand. Furthermore, Mukai and Hashimoto [16] presented that the average hand length of Japanese first-grade elementary school students is 134 ± 3 mm, and they report the widespread use of bamboo and plastic in chopstick production. Hence, the chopsticks of this product were selected for this study.

### Procedure

Data on children’s chopstick handling was collected at participating schools following the acquisition of consent from both schools and parents. A research team consisting of one researcher (NC) and five students from Niigata University of Rehabilitation conducted the study. Each child participant was assigned a dedicated researcher. To minimize potential psychological stress, the tasks were conducted in a normal classroom environment.

Children were instructed to hold chopsticks in their dominant hand for 10 seconds, maintaining a 45-degree supination of the forearm and a slight wrist extension (Fig 1). The opening and closing speed of the chopsticks was set at two times per second, with the opening width adjusted to be parallel or wider than the near and distal chopsticks. A one-minute practice period preceded the actual task. The entire research procedure was converted into a manual and distributed in advance to ensure consistency in measurement methods among researchers. Data collection took approximately 5 minute per child.

### Classification of the method of chopstick handling

In recent years, building upon the foundational classification of Japanese children’s chopstick-holding patterns [24–26], Yokubo et al. [27] classified these patterns into three main categories: four-finger, three-finger, and palm prehension. This classification is based on the number of fingers in contact with the chopsticks during manipulation.

The categories are as follows:

Four-finger prehension: This four-finger method features the upper chopstick held by fingers I, II, and III, while the bottom one rests between fingers I and IV. This allows all four fingers to move the chopsticks with independent flexibility and coordinated operation. In this study, we defined the four-finger prehension grip as the traditional chopstick-holding style.

Three-finger prehension: A chopstick-holding technique utilizing the Ⅰ, Ⅱ, and Ⅲ fingers, these fingers operate the chopsticks with independent and coordinated movements.

Palm prehension: The palm, with minimal finger involvement, primarily drives chopstick movement in palm prehension. This technique is less precise and requires more effort than the other two methods.

For videos demonstrating different chopstick handling techniques, please refer to the supporting information (S2 Appendix). This study used the Yokubo et al. [27] classification, which is designed following the unique culture of Japan. Two researchers (YC and NC) evaluated chopstick-handling methods by separately reviewing videos of participants’ chopstick operations. Researchers reviewed the video together to reach a consensus if they disagreed on their assessments of a participant’s chopstick-handling method.

### Information sheet

Before the testing, all parents were given an information sheet that included questions about basic demographic data, their children’s chopstick handling skills (such as experience with chopstick training and knowledge of chopstick handling), and the Developmental Coordination Disorder Questionnaire (DCDQ).

The DCDQ is a parent-report questionnaire widely used to determine developmental coordination disorders in children aged 5–15 years [28]. The DCDQ is a 15-item scale divided into three factors: control during movement, fine motor/handwriting, and general coordination. Each item is rated on a 5-point Likert scale. It is a reliable and well-validated tool for both research and clinical purposes [29, 30]. In this study, we used the standardized Japanese version of the DCDQ to assess the motor skills of children [31].

### Data analysis

The study began with a descriptive analysis of participants’ demographic characteristics. To identify factors associated with traditional chopstick use, we employed multinomial logistic regression. This statistical method allowed us to predict the likelihood of using different chopstick-holding styles (four-finger, three-finger, or palm prehension). We determined the specific chopstick grip by assessing interrater reliability using the Fleiss kappa coefficient. To account for potential outliers, we used robust maximum likelihood estimation [32]. Missing data were handled using full information maximum likelihood to ensure unbiased results [33]. Our analyses were conducted using statistical software including M-plus (Ver 1.8.10), R (Ver 4.2.0), and Statistical Package for the Social Sciences (Ver 27.0.1.0).

Independent variables, such as age, gender, living arrangements, prior chopstick training experience, and DCDQ scores, were selected based on previous research [13, 14, 34]. To assess the normality of the DCDQ scores, we used the Kolmogorov-Smirnov test. For variables that did not meet the normality assumption, we applied a logarithmic transformation [35]. The odds ratio (OR) and 95% confidence interval were presented, and statistical significance was set at an alpha criterion of p-values of <0.05. The predictive power of the model was evaluated using a pseudo-R-squared statistic [36].

**Fig 3:**
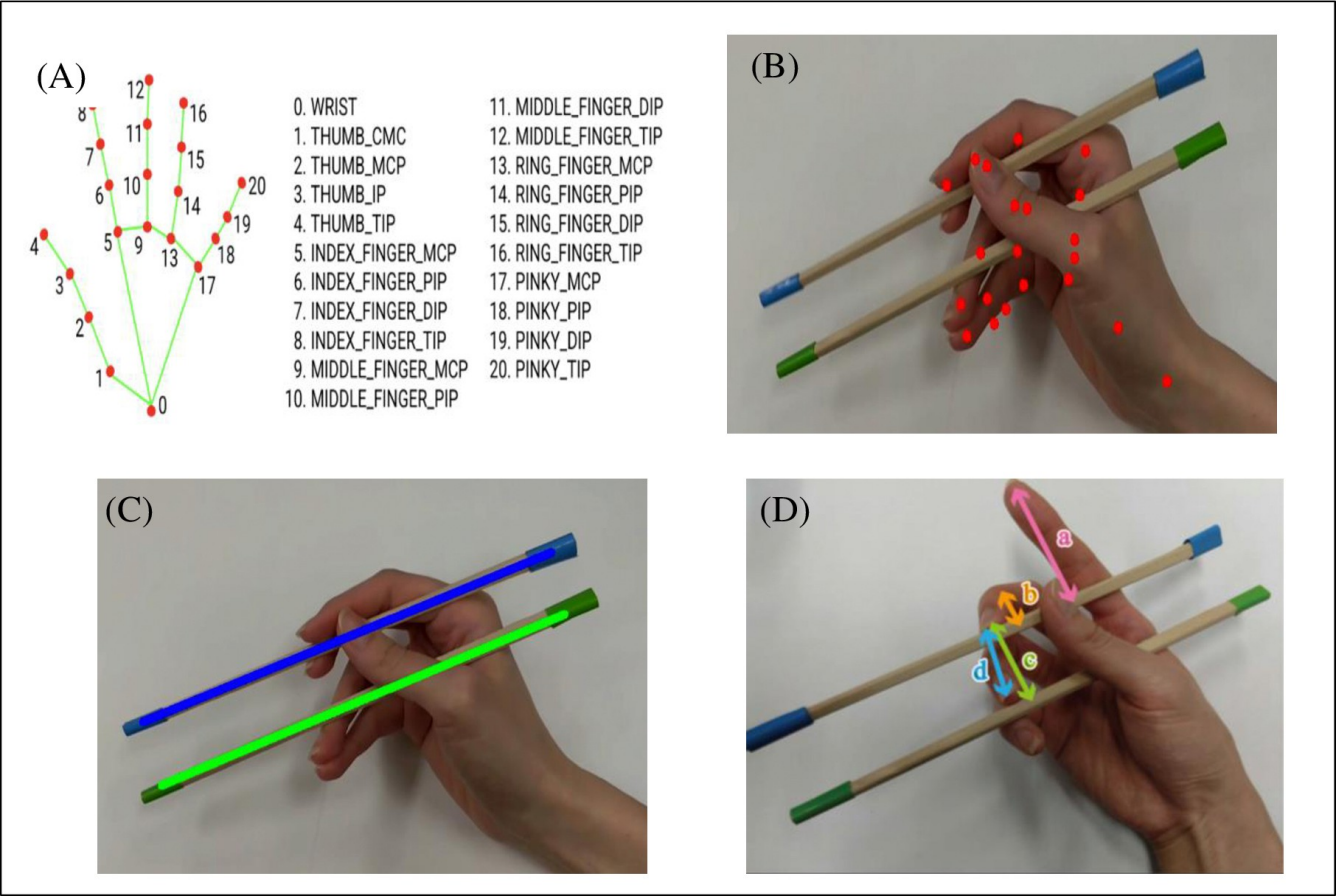
Hand landmarks and chopstick detection. (A) Hand landmarks. (B) Hand landmarks when holding chopsticks. (C) Chopsticks detection. (D) Features used for analysis; a. Vertical distance between the fingertip of finger II and the upper chopstick. b. Vertical distance between the first joint of finger III and the upper chopstick. c. Vertical distance between the fingertip of finger III and the lower chopstick. d. Distance between the fingertip of finger III and the fingertip of finger IV. Images (A) adapted from [37].

**Fig 4:**
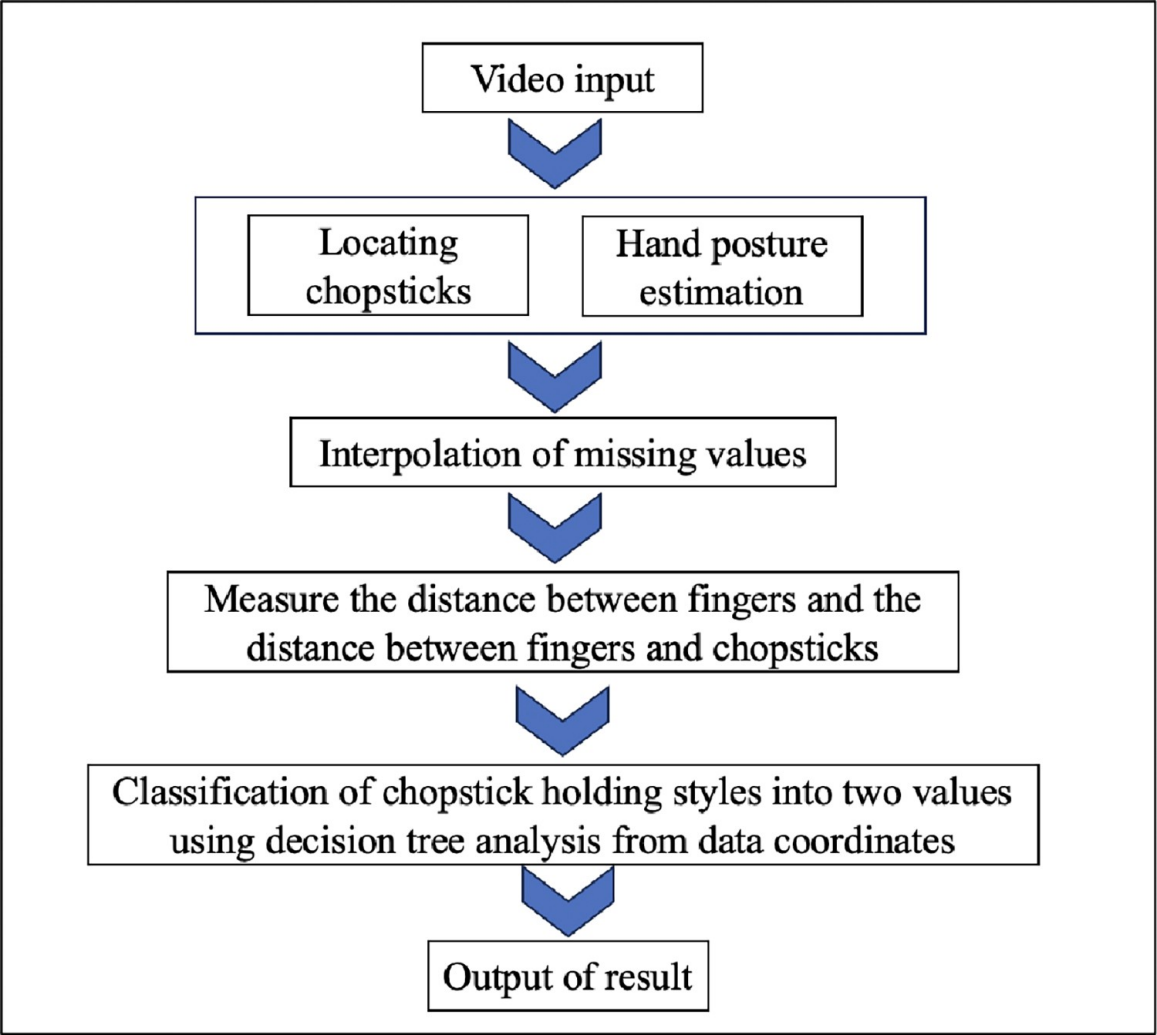
Flow of the video analysis.

Hand landmarks were extracted from chopstick operation videos using MediaPipe, an open-source library developed by Google for hand pose detection. MediaPipe efficiently acquired 21 landmarks from each hand, including 4 points per wrist and each finger, using a single camera [37] (Fig 3). This was particularly advantageous in our study, as it avoided the burden of multiple cameras on the children. We analyzed 163 video samples, including 60 with a traditional grip and 103 with a different grip. To balance the dataset, we extracted features and augmented the traditional grip samples by adding scaling and translations. This doubled the number of samples for traditional grips. To address potential errors in hand landmark and chopstick detection, we incorporated data from the previous frame into our analysis. We used 21 hand landmark coordinates, the positions of the upper and lower chopsticks, and specific distances between fingers and chopsticks as features. In addition, the position of the chopsticks was determined by using image processing to identify the color of the tape attached to the chopsticks. The correctness of the model was evaluated based on interrater agreement among researchers. A decision tree algorithm was employed, with weights assigned to features based on their importance for traditional and nontraditional grips. Time series analysis was conducted using the CO_f1ecac index, which captures the autocorrelation in time series data [38]. The results of this analysis were used to classify the chopstick grip into traditional and nontraditional categories. The analysis process is depicted in Fig 4.

## Results

### Classification of the chopstick-handling method

The classification of chopstick handling demonstrated high interrater reliability (Fleiss’ kappa = 0.966), and the final results were as follows: 64 (38.8%) participants were classified as four-finger prehension, 49 (29.7%) as three-finger prehension, 20 (12.1%) as palm prehension, and 32 (19.4%) as others. Of the 64 participants classified as having four-finger prehension, only 16 (9.7%) could correctly operate chopsticks. The remaining 48 participants experienced instability during chopstick manipulation due to the finger Ⅳ slipping from the lower chopstick or the fingerⅡ and finger Ⅲ slipping from the upper chopstick. Many participants classified as others demonstrated a pattern similar to palm prehension, but the contact points between the chopsticks and fingers were different. This study considered palm prehension and others as a single group.

### Mothers’ age and chopstick teaching experience

The mothers’ mean age was 37.88 ± 6.11 years (range: 26–49). Among them, 136 (82.4%) reported having experience teaching their children how to use chopsticks, 27 (16.4%) reported not having such experience, and 2 did not respond. Additionally, 152 (92.1%) reported knowing how to use traditional chopsticks, 12 (7.3%) reported not knowing, and 1 did not respond. Of the total number of mothers surveyed, 117 (70.9%) reported their ability to use traditional chopsticks, while 47 (28.5%) reported inability and 1 did not respond.

### DCDQ

Table 1 presents the results of the DCDQ for Japanese children. The mean total score was 51.25, with subscores of 20.24 for control during movement, 14.44 for fine motor/handwriting, and 16.57 for general coordination. The 6- and 7-year-old groups exhibited total scores of 50.20 and 53.50, respectively. Boys and girls demonstrated mean scores of 50.42 and 51.93, respectively.

**Table 1:** Results of the DCDQ, mean ± SD.

	Total	Age		Sex	
	(n = 165)	6 age (n = 114)	7 age (n = 51)	Boy (n = 75)	Girl (n = 90)
DCDQ					
Control during movement	20.24 ± 4.32	19.71 ± 4.07	21.38 ± 4.65	20.90 ± 4.62	19.70 ± 4.00
Fine motor/handwriting	14.44 ± 3.49	14.33 ± 3.50	14.68 ± 3.47	13.42 ± 3.61	15.28 ± 3.16
General coordination	16.57 ± 4.01	16.16 ± 3.87	17.44 ± 4.20	16.10 ± 4.05	16.95 ± 3.95
Total	51.25 ± 9.93	50.20 ± 9.55	53.50 ± 10.45	50.42 ± 10.66	51.93 ± 9.30

DCDQ: Developmental Coordination Disorder Questionnaire

The DCDQ results are presented as sums of raw scores.

Chopstick handling methods were classified according to Ohkubo et al.’s three-stage classification: four-finger prehension, three-finger prehension, and palm prehension. Additionally, palm prehension includes those that do not fall into any of the three classifications.

### Factors affecting traditional chopstick-handling

Researchers identified the determinants of traditional chopstick handling using multinomial logistic regression analysis, with four-finger prehension as the reference category group (Table 2). The dependent variable was the total DCDQ score, which was normally distributed by the Kolmogorov–Smirnov test; therefore, logarithmic transformation was not performed.

**Table 2:** Determinants of traditional chopstick holding.

	Three-finger prehension vs four-finger prehension		Palm prehension vs four-finger prehension	
	OR (95%CI)	*p*	OR (95%CI)	*p*
Age	0.49 (0.18, 1.30)	0.15	0.33 (0.12, 0.97)	0.043
Sex	1.35 (0.55, 3.32)	0.51	0.96 (0.40, 2.34)	0.933
Grandparent-grandchild cohabitation	0.15 (0.06, 0.37)	<0.001	0.12 (0.05, 0.31)	<0.001
Prior experience in chopstick training	0.59 (0.19, 1.76)	0.34	1.66 (0.49, 5.64)	0.42
DCDQ total score	1.01 (0.96, 1.05)	0.82	0.99 (0.95, 1.04)	0.73

Dummy coding (Sex: boy = 1 and girl = 0; grandparent-grandchild cohabitation: yes = 1 and no = 0; prior experience in chopstick training: yes = 1, no = 0); OR: odds ratio; CI: confidence interval.

Only grandparent–grandchild cohabitation demonstrated significance when comparing three-finger prehension with four-finger prehension (OR = 0.15, p < 0.001). Age (OR = 0.33, p = 0.043) and grandparent–grandchild cohabitation (OR = 0.12, p < 0.001) were significant when comparing palm prehension with four-finger prehension. The Nagelkerke R2 value was 0.23.

### Classifying chopsticks into traditional and nontraditional categories

**Fig 5:**
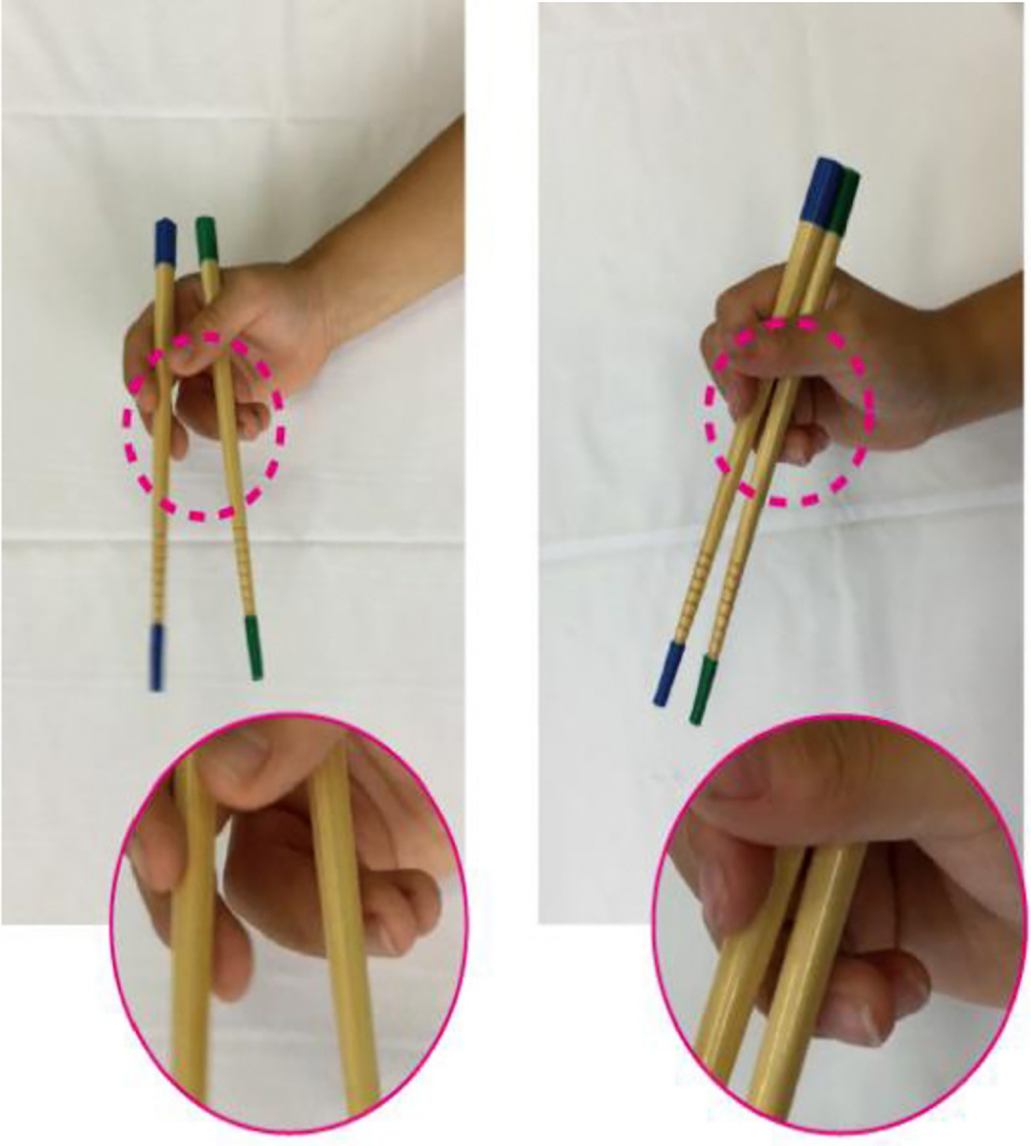
Differences in precision in how to hold chopsticks. The left figure shows the large movements of fingers III and IV during chopstick opening and closing, whereas the right figure shows the small movements of fingers III and IV.

The use of MediaPipe resulted in a high accuracy rate of 0.851, precision of 0.833, and recall of 0.882 (F = 0.862) for identifying whether children’s chopstick handling is traditional or not. The accuracy was higher when the movements of the fingers Ⅲ and Ⅳwere large during chopstick opening and closing operations. However, judging the grip was difficult when the movements of fingers III and IV were small (Fig 5). For video examples of web applications, please refer to the supporting information (S3 Appendix).

## Discussion

### Classification of children’s chopstick grips and the reality of chopstick education

This study classified 80.6% (133 out of 165) of the children into one of the three categories of chopstick grips based on the classification by Yokubo et al. [27]. However, 19.4% (32 children) were similar to the palm prehension type but did not exactly match it. This indicates that similar to children from other countries, Japanese children have a wide variety of chopstick grips [9].

This study revealed a discrepancy between parental self-reported knowledge and practical application of the traditional chopstick grip. Despite a high proportion of parents indicating that they had both learned and taught the correct technique, both generations demonstrated a notable lack of proficiency in chopstick use. A survey conducted by the Japanese Cabinet Office [13] highlighted a significant generational decline in chopstick education, with rates dropping from approximately 80% among those aged 60 and over to 40% among those in their 20s. Given the age profile of parents in our study and the observed correlation between intergenerational cohabitation and children’s chopstick skills, our findings suggest that there is compelling evidence of a discontinuity in the intergenerational transmission of chopstick-related knowledge and skills. Moreover, the heavy reliance on home-based chopstick education, as evidenced by the fact that 90% of such education occurs within families [13], and the limited involvement of educational institutions may have exacerbated this issue. At present, while there are some reports on chopstick education in schools, detailed studies are scarce, and most research on children’s chopstick use relies on surveys [14]. In the future, it will also be increasingly important to conduct qualitative research, such as nationwide surveys on chopstick education in schools and in-depth interviews with parents and children, to gain a deeper understanding of intergenerational and lifestyle factors influencing chopstick skills.

The age factor has been traditionally considered important in hand function [39], therefore, a significant difference was found between palm prehension and four-finger prehension, which have low finger dexterity during chopstick operation. However, the results of the DCDQ did not demonstrate a significant difference between chopstick grip and children’s finger dexterity. Traditionally, a relationship exists between chopstick grip and finger dexterity, but Hatta and Kawakami [4] concluded that dexterity is not related to traditional or nontraditional grip. This indicates that the acquisition of the traditional chopstick grip may be more dependent on parents’ ability to properly evaluate and teach their children’s chopstick grip than on children’s finger dexterity. This study only evaluated children’s dexterity from the perspective of the DCDQ, thus continuous investigation on how finger dexterity affects the traditional chopstick grip is necessary. Furthermore, additional research is warranted to investigate the potential impact of the influx of Western cuisine on the frequency of chopstick use and the development of finger dexterity in Japanese individuals.

### Analysis of the traditional chopstick grip using the hand pose estimation model

Traditional evaluations of children’s chopstick skills often rely on a limited set of predetermined grip types [24–27]. However, these methods may overlook subtle deficiencies, such as insufficient lower chopstick fixation or upper chopstick displacement, that can compromise performance in real-world eating situations. To address these limitations, this study employed a hand pose estimation model to analyze video data of children using chopsticks. The results demonstrated the feasibility of accurately classifying children’s chopstick skills using a binary classification. By incorporating chopstick recognition, this study extends previous research on hand gesture recognition [40–42] and provides a foundation for developing user-friendly web applications to assess chopstick skills. While this study focused on traditional chopstick grips, a comprehensive comparison with other grip types remains to be conducted. Therefore, future research should explore the optimal grip for children. Furthermore, it is anticipated that the development of this application will contribute to the systematization of knowledge and skills related to chopstick use, potentially by integrating it into school curricula and digital learning tools. This is expected to complement existing home-based chopstick education.

In this study, data were collected using chopsticks provided by regular classrooms in Murakami City. However, the generalizability of the findings is limited by factors such as the participants’ age, disability status, geographic location, and the involvement of parents in data collection. Additionally, the type of chopsticks used may influence the results. While the hand pose estimation model achieved high accuracy in analyzing the traditional chopstick grip, it was susceptible to misrecognition when the movements of fingers III and IV during chopstick opening and closing were subtle. To address these limitations, future studies should consider a more diverse sample population and a wider range of chopstick types. To further enhance user convenience, we can also explore methods to improve the accuracy of chopstick and finger analysis even without color-coded tape. The successful application of the hand pose estimation model opens up possibilities for the development of a web application to assess chopstick skills. Such an app could provide personalized feedback to users, enabling them to improve their chopstick technique. However, several practical considerations must be addressed before implementing such a web application. For example, we need to consider whether high-accuracy detection can be achieved from various camera angles, and whether the system can provide results instantaneously from measurement.

## Conclusion

This study aimed to investigate the current status of the chopstick grip in first-grade elementary school students (6–7 years old) and to identify factors related to the traditional grip. We also proposed a method to objectively determine children’s traditional chopstick grip using a clinically applicable hand pose estimation model. The results revealed that approximately 80% of children’s chopstick grips were classified into three types: four-finger prehension, three-finger prehension, and palm prehension according to the classification of Yokubo et al, but the remaining 20% did not belong to any of these types. Additionally, >80% of parents had instructed their children on how to hold chopsticks, but only 9.7% had acquired a traditional grip. The factors that affected traditional grip were grandparent–grandchild cohabitation and age. These results reveal that traditional grip involves the lack of transmission of chopstick education from grandparents to parents and that parents themselves may not have sufficient knowledge about chopsticks. The method of objectively identifying children’s traditional chopstick grip using a hand pose estimation model was confirmed to have high accuracy. In the future, we will begin developing a web application that can be widely used in educational settings and households.

## Supporting information

S1 Appendix Raw data. (XLSX)

S2 Appendix Types of chopstick grips (example). (MP4)

S3 Appendix Web application (example). (MP4)
